# Damage-associated molecular patterns in trauma

**DOI:** 10.1007/s00068-019-01235-w

**Published:** 2019-10-14

**Authors:** Borna Relja, Walter Gottlieb Land

**Affiliations:** 1 Experimental Radiology, Department of Radiology and Nuclear Medicine, Otto von Guericke University Magdeburg, Magdeburg, Germany; 2 Department of Trauma, Hand and Reconstructive Surgery, University Hospital Frankfurt, Goethe University Frankfurt am Main, 60590 Frankfurt, Germany; 3 Molecular ImmunoRheumatology, INSERM UMR_S1109, Laboratory of Excellence Transplantex, University of Strasbourg, Strasbourg, France

**Keywords:** DAMP, SAMP, Danger, Inflammation, Trauma

## Abstract

In 1994, the “danger model” argued that adaptive immune responses are driven rather by molecules released upon tissue damage than by the recognition of “strange” molecules. Thus, an alternative to the “self versus non-self recognition model” has been provided. The model, which suggests that the immune system discriminates dangerous from safe molecules, has established the basis for the future designation of damage-associated molecular patterns (DAMPs), a term that was coined by Walter G. Land, Seong, and Matzinger. The pathological importance of DAMPs is barely somewhere else evident as in the posttraumatic or post-surgical inflammation and regeneration. Since DAMPs have been identified to trigger specific immune responses and inflammation, which is not necessarily detrimental but also regenerative, it still remains difficult to describe their “friend or foe” role in the posttraumatic immunogenicity and healing process. DAMPs can be used as biomarkers to indicate and/or to monitor a disease or injury severity, but they also may serve as clinically applicable parameters for optimized indication of the timing for, i.e., secondary surgeries. While experimental studies allow the detection of these biomarkers on different levels including cellular, tissue, and circulatory milieu, this is not always easily transferable to the human situation. Thus, in this review, we focus on the recent literature dealing with the pathophysiological importance of DAMPs after traumatic injury. Since dysregulated inflammation in traumatized patients always implies disturbed resolution of inflammation, so-called model of suppressing/inhibiting inducible DAMPs (SAMPs) will be very briefly introduced. Thus, an update on this topic in the field of trauma will be provided.

## Introduction

Traumatic injury is one of the major contributors to worldwide mortality as well as one of the world’s most relevant but neglected health concerns [1, 2]. Multiply injured or polytraumatized patients most frequently die either immediately or early within only of a few hours after the traumatic insult due to their injury severity, severe traumatic brain injuries (TBI), and/or exsanguination, or in the later post-injury phase due to inflammation-related complications, which affect the immune system homeostasis, resulting in, e.g., sepsis, septic shock, or multiple organ failure (MOF) [2–6]. Adjacent to physical factors, which are intensely treated in the intensive care units (ICU), in case of survival, the multiply injured patient may suffer from serious impairments in cognitive, psychological, or psychosocial issues [7–9].

**Fig. 1:**
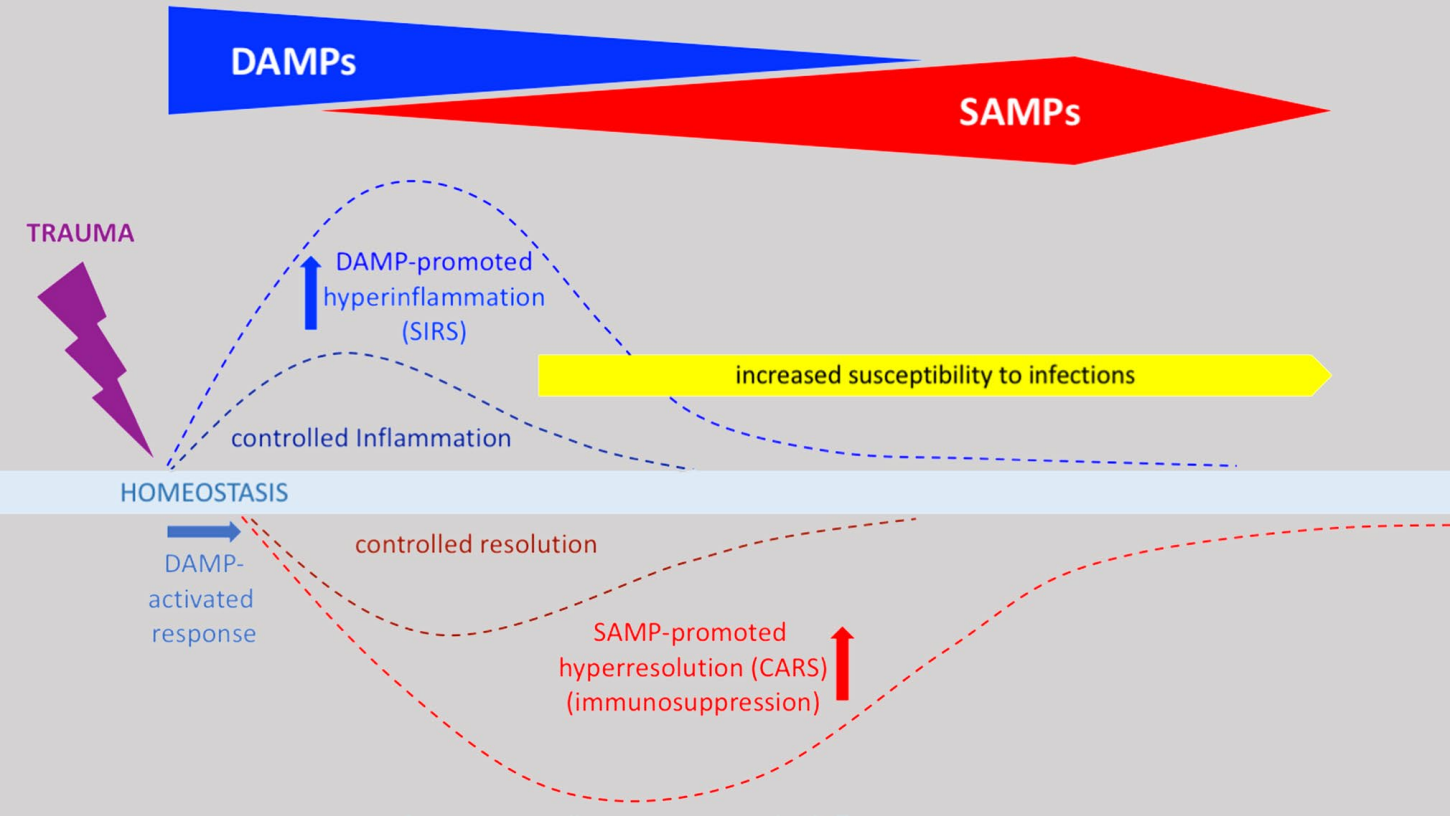
Brief concept of posttraumatic inflammatory response with the involved damage-associated molecular pattern (DAMPs) and suppressing/inhibiting inducible DAMPs (SAMPs) and their release/activation as well as hypothesized kinetics upon trauma. CARS: compensatory anti-inflammatory response syndrome; SIRS: systemic inflammatory response syndrome

Over the last decades, several experimental (poly)trauma models which combine different injury patterns have been developed and applied to study and understand the basic pathophysiology of the posttraumatic sequelae in polytraumatized patients. While the primary impact can only be addressed by injury prevention, the later detrimental sequalae may be prevented by an abrogation of the posttraumatic course [10, 11]. The typical pathophysiological course of traumatized patients under ICU treatment is characterized by a systemic inflammatory response to severe injury that begins immediately after trauma, and which involves complex interactions across the hemostatic, inflammatory, endocrine, and neurological systems [3, 12, 13]. Modern notions hold that this clinically observed so-called systemic inflammatory response syndrome (SIRS) reflects an uncontrolled overshooting systemic innate immune response caused by polytrauma-induced emission of large amounts of so-called damage-associated molecular patterns (DAMPs) which circulate systemically to affect the whole body of the patient [3, 12–15]. The term DAMP was coined in 2003 by Land [15]. And it is apparently the DAMPs which lead to the catastrophe of the sterile hyperinflammatory, sepsis-like, life-threatening SIRS, which is most often associated with organ failure, MOF, or multiple organ dysfunction syndrome (MODS) [12, 13, 16]. Typically, this DAMP-induced systemic cytokine/chemokine-mediated hyperinflammatory response to severe trauma is associated with an intense and long-lasting counterbalancing compensatory anti-inflammatory response syndrome (CARS) resulting in posttraumatic immunosuppression (IS) [3, 5, 17–19]. CARS can be regarded as a disorder caused by a hyper-resolution of hyperinflammation in terms of a mirror-imaged counter-regulation of SIRS. Thus, for several decades research on posttraumatic complications was based on a biphasic posttraumatic inflammation model. The central pathophysiological principle there was to find the balance between a qualitative defense against invasive putative pathogens and additionally reducing collateral damage by immune cells [20–22]. Of note, however, instead of a previously assumed biphasic inflammatory response consisting of an initial proinflammatory and a subsequent counterbalancing anti-inflammatory response, hyper-resolving processes are now considered to occur simultaneously with hyperinflammation. There is presumably only a slight delay between initial hyperinflammation and the following hyper-resolution, which is possibly caused by the production of suppressing/inhibiting inducible DAMPs (SAMPs) by already DAMP-activated/initialized responses of innate immune cells [23]. Recently, this novel term accomplishing the DAMPs/SAMPs post-injury response was coined by Land [23]. The posttraumatic course of this inflammatory response is briefly represented in Fig. 1.

## SIRS

The recognition of DAMPs by immune cells initiates an acute hyperinflammatory condition of SIRS that induces physiological changes such as hypothermia or hyperthermia, elevated heart rate, and leukocytosis/leukocytopenia [24, 25]. The uncontrolled or protracted clinical signs of SIRS constitute a risk factor for organ failure, delayed hospital-acquired infections as well as occasionally MOF after trauma [26–28]. The concept of SIRS was introduced in early 1990s and charged with the task of developing an easy-to-apply set of clinical parameters, as well as biomarkers, to improve the early identification of potential candidates for clinical trials and new treatment strategies for sepsis, as well as for the identification of patients at risk of infectious complications, respectively [25]. Thus, since three decades, the knowledge on the complex pathophysiological and posttraumatic inflammatory response contributing to the development of post-injury complications is growing [29–32]. Numerous cellular and humoral proinflammatory mediators including complement factors are markedly elevated in patients suffering from SIRS and associated complications on the other hand. In general, SIRS is classically dominated by overwhelming hyperinflammatory processes which are characterized by elevated circulating levels of multiple powerful cellular and humoral proinflammatory mediators. Of those, notably in the experimental and clinical sepsis-associated SIRS, cytokines (e.g., tumor necrosis factor (TNF), interleukin (IL)-1α, IL-1β, and IL-6), chemokines, growth factors, adhesion molecules, gaseous substances, vasoactive peptides, and cell stress markers have been identified and analyzed [33]. After the concept of SIRS was created, a number of publications emerged heralding the efficacy of SIRS in predicting outcomes after trauma [34, 35]. Accumulating evidence indicates that SIRS associated with MOF is the result of traumatic injury-induced either accidental or regulated cell death such as necroptosis, serving as a robust source for emission of DAMPs, while the impact of apoptosis may be presumably less significant here [36–39]. Today, it is widely accepted that DAMPs are the key predominant molecules which trigger the biological host response to trauma including SIRS, by activating and recruiting effector cells including antigen-presenting cells of the immune system [12, 40–46]. Thus, basically, every DAMP proven to promote efferent proinflammatory pathways can be considered to be involved in the development of SIRS. In 2012, in accordance with the newly recognized “sterile SIRS” as a severe complication form in polytraumatized patients, first clues to a critical role of DAMPs in the promotion of those hyperinflammatory responses have been reported [47]. Among those molecules, endogenous DAMPs including high-mobility group box (HMGB) protein 1, heat shock proteins (HSPs), certain members of the S100A family, histones, free nucleic acids, members of the IL-1 cytokine as well as complement family as *bona fide* intracellular effectors, which upon abundant rapid release alert the environment about cell stress and danger, have already been reported to activate the posttraumatic innate immune responses and drive organ dysfunction(s) after trauma [12, 13, 46, 48].

Although a large number of endogenous nuclear or cytosolic DAMPs have been described in the context of the local/systemic posttraumatic and/or noninfectious “sterile” inflammatory response, which represents the key driver of the late occurring post-injury complications and fatal outcome rates, the knowledge on their variety and underlying mechanisms still remains unknown [49–51]. In particular, it is unknown whether there is a distinct pattern of DAMPs emission that may be helpful in differentiating between, i.e., sterile and infectious (bacterial or viral) sepsis form. The knowledge on posttraumatic DAMPs pattern may allow to further define the exact roles of DAMPs in hyperinflammation and develop therapeutic inhibitors targeting specific DAMPs to improve outcomes. Thus, some of most critically involved DAMPs in both sterile and infectious posttraumatic disorders will be briefly described in this review.

## CARS and SAMPs

The recognition of an overshooting SIRS which may be associated with septic complications has provided several attractive targets for new anti-inflammatory drugs which were designed to prevent further propagation of inflammation in past. Yet, nowadays, treatment of sepsis in critically ill patients is mainly based on the prompt intravenous administration of adequate antimicrobial agents and support of organ functions. For the last four decades, in theory, many different targets for treatment were designed to inhibit, bind, or neutralize the proinflammatory mediators that were assumed to be responsible for the network of events that culminated in detrimental clinical outcomes. Disappointingly, however, targeted clinical trials have shown that most of these agents failed to cure the septic disease [52–56]. Even the promising US FDA-approved drug, recombinant human-activated protein C or drotrecogin-α failed in clinical trials and showed similar effects as placebo. Intriguingly, these observations paved the way to another typical symptomatic feature of the disorder: the CARS [24]. The high susceptibility to secondary infections after the initial phase of sepsis or after trauma has been attributable to CARS causing the posttraumatic IS [17, 19, 57]. Interestingly, for a long time, intensive care physicians have observed that patients with SIRS were prone to severe, sometimes lethal infectious diseases. However, in search of the causes during the 1970s and 1980s, researchers found that many of those patients had acquired an impaired immune state characterized by various markers of IS. The concept was actually underlined from the beginning as the treatment strategies with anti-inflammatory agents failed in clinical trials on septic patients.

The phenomenon of impaired immunity that was frequently observed in posttraumatic as well as in septic conditions essentially reverses many of the typical events encountered in SIRS. However, research over the last several decades uncovered the anti-inflammatory and immunosuppressive mechanisms that govern the clinical post-injury disorders. Initially, IS corresponds to a homeostatic phenomenon to avoid the remote organ injuries caused by the early SIRS; however, it becomes deleterious when it persists, rendering patients prone to secondary infections [17, 58]. Nowadays, CARS is not regarded as part of a biphasic inflammatory response consisting of an initial SIRS with a subsequent CARS. It became evident that the hyper-resolving processes of inflammation (CARS, IS) occur nearly in parallel with the hyperinflammatory response, but also that they can exist separately from the SIRS [28, 59]. Moreover, CARS is not only a simplified anti-inflammatory process, but rather a disorder caused by a hyper-resolution of inflammation.

Remarkably, next to alterations of the innate immune system, posttraumatic IS has been closely linked to modulations of the adaptive immune system. Here, among others, i.e., a shift from a T-helper cell type (Th)1- to a Th2-mediated immune response, T cell anergy in traumatized patients, suppression of T cell effector functions, modulated frequency of T regulatory cells have been observed [19, 21, 22, 60–64]. This posttraumatic response suggests that in reverse with the proinflammatory action of DAMPs in SIRS, SAMP triggers the proresolving pathways in CARS and IS. Besides activating DAMPs, potentially suppressing DAMPs, that is SAMPs, are mainly produced by activated leukocytes and macrophages upon stress and injury, which—in parallel to proinflammatory responses, convey anti-inflammatory processes and shape immunosuppressive responses. In 2015, it was shown that the gene expression of proresolving lipid mediator pathways was closely associated with clinical outcomes in traumatized patients [65]. Patients with uncomplicated recoveries had differential and higher resolving scores [65]. The SAMPs prostaglandin E2 or AnxA1 plays significant roles in proresolving CARS [66–68]. Accordingly, DAMPs and SAMPs constitute valuable tools in diagnosis and therapy of severely injured and/or infected patients that are expected to find their way into clinical routine at the ICUs. Hyper-resolution with the following IS may be the most critical factor for the immune status of patients with either sterile or infectious SIRS. Thus, here as well, measurement of DAMPs with their pattern, but also continuous determination of SAMPs with their pattern, respectively, may be used as hospital admission criteria for decisions supporting further diagnostic, prognostic, and therapeutic modalities. Although a closer look into both DAMPs and SAMPs is necessary to understand the posttraumatic response, in the underlying review only DAMPs will be addressed.

## DAMPS

DAMPs are released from a damaged or diseased cell, and upon their release, they can stimulate a sterile immune or inflammatory response [13, 49, 69]. Across the tree of life, DAMP-induced immune responses serve as defense strategies aimed at maintaining and restoring homeostasis. However, despite their initially beneficial character, when dysregulated, uncontrolled, and exaggerated, the inflammatory and tissue repairing processes may become pathogenic and can lead to serious pathologies, i.e., sepsis, cardiovascular diseases, neurodegenerative diseases, diabetes, obesity, and asthma, as well as classic inflammatory diseases. Notably in the last two decades, the biological host response to trauma, which initially has been characterized by massive cytokine release as well as the activation and recruitment of effector cells including antigen-presenting cells, has further employed a large number of both microbial and host alarmins [12, 40–44]. In general, on the biochemical level, the posttraumatic immune response is not only activated by foreign non-self material, but includes also endogenous factors, DAMPs, which are released from necrotic or physiologically “stressed” cells, with the aim of initiating and recruiting effector cells of the immune system [70–72]. A large number of those endogenous nuclear or cytosolic triggers have been described to initiate and perpetuate the systemic posttraumatic and/or noninfectious inflammatory response; however, the knowledge on their precise role still remains unknown [13, 49–51]. They signal “danger” to the host and trigger an inflammatory response, which in physiological healing leads over to tissue regeneration, but when dysregulated can cause pathologic inflammation and related disorders.

In the pathology of severe trauma, trauma-induced generation/emission of DAMPs can refer to cell-intrinsic modified DAMPs such as cell-free deoxyribonucleic acid cfDNA or DAMPs passively released from dying cells. Thus, any kind of traumatic cell stress/tissue injury provoked by mechanical trauma (e.g., blow, crush, penetrating wound, or surgical procedures usually related to fracture, hemorrhage, and/or infections), thermal trauma, or metabolic trauma such as promoted by ischemia/reperfusion injury, acidosis, hypoxia/hypoxemia, provokes a DAMP release [23]. Clinically, SIRS is characterized by local and systemic release of proinflammatory cytokines produced by DAMP-activated innate immune cells including IL-1β, TNF, and IL-6, arachidonic acid metabolites, proteins of the contact phase and coagulation systems, and complement factors as well as hormonal mediators [3, 34, 73, 74]. Among other factors, proinflammatory cytokines also activate coagulation pathways, whereby enhanced thrombin formation increases fibrinogen cleavage and reduces fibrin monomers from polymerizing to form stable fibrin clots (hypercoagulability), which is why fibrinogen depletion is observed [75]. Disseminated intravascular coagulation (DIC) is a heterogeneous group of disorders, which manifests as a spectrum of hemorrhage and thrombosis complicating many primary conditions including, i.e., sepsis and trauma [76]. Although its pathophysiology is complex, there is growing evidence that DAMPs emitted in excess play a crucial role in the pathogenesis of DIC [76]. Here, i.e., cfDNA, also known as extracellular DNA, which can be released by various host cells including neutrophils, macrophages, eosinophils, and tumor cells, as well as by certain strains of bacteria plays a significant role [77–82]. Elevated levels of cfDNA have been found in various pathologic conditions including trauma [83, 84]. Interestingly, cfDNA that appears to exert both pro- and anti-fibrinolytic effects is a good predictor of trauma patient’s outcome in the ICU [84]. Activation of neutrophils with microbial or inflammatory stimuli results in the release of neutrophil extracellular traps (NETs) [77]. Upon cell death or specific cell activation of hematopoietic and parenchymal cells, extracellular cfDNA as well as DNA-binding proteins (e.g., histones and HMGB1) are released into circulation [13]. Those DNA-binding proteins are also strongly procoagulant and are involved in the pathogenesis of DIC [76, 85]. Another factor that can be directly influenced by DAMPs and contribute to DIC is the cellular migratory behavior with barrier loss. DAMP-induced endothelial expression of adhesion molecules facilitates leukocyte adhesion, promoting extravasation of leukocytes from the circulation into damaged tissue [86]. Mitochondrial (mt) DAMPs like mitochondrial DNA (mtDNA) and peptides appear in the blood after injury or shock, activate human leukocytes and might contribute to increased endothelial permeability during systemic inflammation [87]. The various DAMP motifs from mitochondria can act on endothelia and/or leukocytes via multiple pathways by enhancing leukocyte adherence to endothelial cells, activating cell–cell interactions, and subsequently increasing systemic endothelial permeability [87]. Mitochondrial DAMPs may be important therapeutic targets in conditions where inflammation pathologically increases endothelial permeability [87]. The mechanisms of a concerted action between endothelial cells and leukocytes, endothelial cell damage, leukocytes extravasation, microcirculatory disturbances, or DIC frequently lead to cell loss of parenchymal cells with the following organ failure. Increased systemic levels of DAMPs have been positively correlated with mortality and morbidity of patients or animals with trauma or surgical insults, while blocking or neutralizing DAMPs with specific small molecules or antibodies ameliorated sepsis and SOF in vivo [88]. As briefly introduced above, the clinical manifestation of severe multiple tissue injury including burn damage is characterized by a systemic cytokine/chemokine-induced hyperinflammatory response resulting in SIRS which is accomplished by an intense and long-lasting CARS associated with posttraumatic immunosuppression that predisposes patients to infectious sepsis and MOF [3, 17, 19, 57]. For example, in a murine burn injury model, IL-10 levels were significantly increased at 24 h, 7 days, and 21 days post-injury as compared with IL-10 levels in sham mice [89]. Thus, accumulating evidence suggests that clinically observed infectious complications are associated with high susceptibility of the patient to secondary bacterial, fungal, and viral infections and that this state is mediated by suppressing DAMPs by SAMPs which control homeostasis following injury. Yet, in the following, a closer look at the role of exemplary DAMPs in traumatic setting is taken. Since DAMPs are a culmination of highly heterogenous mediators, it is nearly impossible to choose the most important ones. Thus, in this review, we focus on the most prominent DAMPs involved in the posttraumatic pathophysiology, but other DAMPs of equal importance, which have been neglected here, may be addressed in further studies. This review sought to primarily describe the exemplary variety of DAMPs in their preclinical and clinical context.

### High-mobility group box protein

HMGB1 as one of best studied DAMPs is a non-histone chromatin nuclear peptide that acts as a DNA chaperon, which is involved in binding of proteins and in DNA transcription, replication, but also repair [90, 91]. HMGB1 is composed of two positively charged DNA-binding motifs and a C-terminal acidic tail [92]. Nearby, all cells constitutively express HMGB1. It is located in the nucleus, but it will be released either passively after necrotic cell death, or secreted actively by living cells undergoing stress in response to angiogenic and inflammatory triggers [93–96]. HMGB1 acts as an endogenous ligand of toll-like receptors (TLRs) 2, 4, 9 and receptor for advanced glycation end products (RAGE), thereby inducing nuclear factor “kappa-light-chain-enhancer” of activated B cells (NF-κB) activation [97, 98]. Although the mechanisms of HMGB1 secretion are elusive, it has been shown that its processing via inflammasomes is involved [99].

The innate immune stimulatory activity of HMGB1 has been regulated by the redox state of cysteine residues C23, C45, and C106 [100]. Interestingly, while a reduction in cysteine residues makes HMGB1 rather a chemoattractant than a cytokine inducer, oxidization of the cysteine residues via reactive oxygen species abrogates both activities of HMGB1 [101]. Thus, it remains elusive, if HMGB1 levels indeed reflect its innate immunity-inducing potential, since the HMGB1 levels do not necessarily reflect the complexity of inflammatory complications with underlying downstream signaling.

Under physiological conditions, HMGB1 is predominantly anchored inside the nucleus via nuclear localization sites and accordingly its binding with nuclear cargo carrier proteins [102, 103]. Systemic levels of HMGB1 increased within 30 min after severe trauma in patients [104] although its regulated activity HMGB1 levels correlated with injury severity, tissue hypoperfusion, early posttraumatic coagulopathy, hyperfibrinolysis, with complement activation and with SIRS [104]. Early increase in systemic HMGB1 levels indicated patients who developed acute lung injury (ALI) or acute renal failure in the later posttraumatic course [104]. Increased levels of HMGB1 were found to stratify non-survivors from trauma as compared to survivors [104]. Deng et al. [103, 105] have recently demonstrated that hepatocytes are the main source of circulating HMGB1 in LPS-induced endotoxemia and cecal ligation and puncture (CLP)-induced polymicrobial sepsis, as well as hemorrhagic shock. They have demonstrated that cell-specific depletion of HMGB1 in hepatocytes dramatically reduced circulating HMGB1 level and conferred protection from sepsis lethality in mice [105]. With regard to trauma, Levy et al. [106] have demonstrated transient elevations in systemic HMGB1 levels within 1 h post bilateral femur fracture. Interestingly, they have shown that after trauma, treatment with neutralizing antibodies to HMGB1 lowered systemic IL-6 and IL-10 levels as compared to controls treated with nonimmune control antibody [106]. Similarly to the above-mentioned study, with regard to hepatic changes, HMGB1 neutralization decreased serum alanine aminotransferase levels, and hepatic as well as gut mucosal NF-κB DNA binding [106]. The authors conclude that HMGB1, more precisely the TLR4-HMGB1 pathway, constitutes as an early mediator of systemic inflammation and end-organ injury after peripheral tissue injury after trauma. In line with these observations, it has been demonstrated in vivo that HMGB1 levels significantly increased in muscle 12 h after crush injury [107]. As already indicated, HMGB1 neutralization by an antibody reduced the cellular apoptosis in the renal cortex, thus indicating a positive feedback cycle [107]. In a systematic review on the role of HMGB1 danger signaling in TBI, HMGB1 was found to be released from damaged neurons, and furthermore, it was elevated in patient’s serum and cerebrospinal fluid (CSF) [108]. Moreover, HMGB1 increased in CSF associated with neuronal death in subarachnoid hemorrhage [109, 110]. The elaborated studies show that HMGB1 may serve as a prognostic biomarker and therapeutic target in patients with TBI [108]. Adjacent to traumatic injury, it was demonstrated before that HMGB1 plays an important role in the initiation and propagation of inflammation and organ injury under conditions of sterile inflammation which involve ischemic processes [111–113]. Also in patients with ventilator-associated pneumonia during mechanical ventilation increased HMGB1 levels in bronchoalveolar lavage fluid (BAL) were detected [114]. In vivo models have demonstrated that intratracheal or intranasal administration of HMGB1 caused ALI, which has been reflected by enhanced acute inflammatory injury to the lungs, neutrophil accumulation, development of lung edema, and increased pulmonary production of IL-1β, TNFα, and macrophage-inflammatory protein (MIP)-2 [115, 116]. Also in different ischemic heart diseases a positive correlation between increased HMGB1 levels and worsen ventricular function upon ischemia/reperfusion injury has been demonstrated [117]. As briefly described in Introduction, DAMPs are playing a role in coagulation processes. HMGB1 itself is also a potent procoagulant that can directly stimulate and recruit platelets through TLR4 and RAGE [88, 118, 119]. Mice with HMGB1-deficient platelets exhibited increased bleeding times and reduced thrombus formation, platelet aggregation, inflammation, and organ damage during experimental trauma/hemorrhagic shock [118]. Extracellular HMGB1 coordinates numerous cellular functions, including migration, chemotaxis, activation, maturation, and proliferation but also the redox status of its target cells [120–123]. However, the binding of HMGB1 to its receptors can be potentiated by binding to a whole variety of other factors, including PAMP and cytokines [123]. Thus, in summary, HMGB1 is an important pathogenic factor of inflammatory and thrombotic complications.

### Interleukin-1

Some of the numerous DAMPs are cytokines, small messenger molecules, which are produced, activated, and released upon trauma [124]. In 1974, Dinarello [125] discovered the first cytokines IL-1α and IL-1β as members of the IL-1 family. IL-1α and IL-1β are encoded by different genes; however, they can be bound by the same IL-1 receptor (IL-1R) [125]. IL-1α has an higher affinity for IL-1R1 and IL-1β for the soluble IL-1R2 [126].

In contrast to IL-1β, IL-1α is constitutively expressed mainly in resting nonhematopoietic cells, which line the gastrointestinal tract, liver, kidney, and skin, but it can also be expressed in most cells and, furthermore, can be biologically active in its full-length form without its previous processing through inflammasomes, as it is mandatory for IL-1β activity [127–129]. IL-1α and IL-33 are expressed in their precursor forms in the nucleus of various hematopoietic cells, and these nuclear proteins play important roles in the regulation of gene expression [130]. Since IL-1α does not contain a secretory signal peptide, it is released into extracellular space through either non-canonical vesicular secretion pathway or passive necrotic release [130]. The release of IL-1α into the extracellular space in stimulated cells occurs after processing of the membrane-bound IL-1α by the membrane-bound calcium-dependent cysteine protease calpain [130–133]. Upon extracellular IL-1α interaction with ubiquitously expressed IL-1R1 and IL-1 receptor accessory protein downstream signaling proteins, such as myeloid differentiation primary response gene 88 (MyD88) and IL-1 receptor-activated protein kinase 4, an inflammatory response is induced [134]. In general, members of the IL-1 family induce similar signaling cascades in their target cells via mitogen-activated protein kinase (MAPK) or NF-κB pathways [135]. IL-1α as a dual function protein is a proinflammatory activator of transcription as chromatin-associated protein, and above that, it is acting as a cytokine [136–138]. The latter is exerting its function after being released from apoptotic or necrotic cells, thereby alerting the immune system to tissue damage [139].

Thus, IL-1α has been considered a potential pathogenic factor involved in the development and progression of several disease including diabetes, inflammatory bowel disease, myocardial inflammation, and cancer [140–143]. Interleukin-1 can initiate many important immunological responses such as fever, prostaglandin synthesis, mobilization of neutrophils into tissues, activation of B and T cell lymphocytes, fibroblast proliferation as well as the production of antibodies, collagen, and cytokines [126, 127]. With regard to trauma, only few studies on IL-1α are available. Jackman et al. [144] observed a mixed early systemic inflammatory response in trauma patients with elevated levels of IL-6, IL-10, IL-1Ra, macrophage migration inhibitory factor, myeloperoxidase (MPO), monocyte chemotactic protein-1, matrix metalloproteinase-9, and soluble Fas ligand, but also with simultaneously decreased levels of fractalkine, epidermal growth factor, IL-7, IL-9, IL-17, TNFbeta (TNFβ), MIP-1α, macrophage-derived chemokine and notably IL-1α. While in vivo DNA microarray data confirmed this highly complex inflammatory response in lung tissue following blunt chest trauma, interestingly, IL-1α has shown elevated expression levels, which were concomitant with increased levels of other inflammatory and coagulatory proteins, including TNFα receptor, IL-1β, C3, NF-κB, and plasminogen activator [145]. In contrast to the above-mentioned study, increased levels of IL-1 have been associated with the pathogenesis of acute respiratory distress syndrome (ARDS), and subsequent idiopathic pulmonary fibrosis, sarcoidosis, as well as certain other inflammatory diseases [130, 146]. Notably alveolar macrophages (AM) from patients with ARDS were shown to release significantly more IL-1 and IL-1β as compared with controls, indicating that increased IL-1 release by AM may be related to the progression of ALI [146]. Also inflammation caused by necrosis or tissue damage upon ischemia or hypoxia caused by poor oxygen supply has been related to IL-1 [130]. Hypoxia upregulated IL-1α transcription in epithelial cells [147]. Ischemia induced IL-1α release in activated platelets of the brain vasculature, and thereby stimulated endothelial cells to secrete the chemokine CXCL1 and express cell adhesion proteins vascular cell adhesion molecule-1 and intercellular adhesion molecule-1, thus promoting the transendothelial migration of neutrophils which are major contributors to inflammation-mediated brain injury [148]. IL-1α-stimulated proinflammatory cytokine expression in necrotic myocytes from ischemic heart following myocardial infarction has been reported as well [149]. It is known that IL-1α is a significant driver of the proinflammatory mechanisms, which are often linked to chronic inflammatory diseases or cancer [130]. However, only little is known about the role of IL-1α during the post-injury inflammatory response. Thus, uncovering its role in trauma and/or trauma-related pathologies may be promising in regard to specific targeting of IL-1α with clinically approved drugs, i.e., anakinra under pathological conditions.

### Interleukin-33

Interleukin-33, a nuclear alarmin, is the youngest member of the IL-1 family, and it is mainly expressed in the cells which are in contact with the environment including stromal cells, such as endothelial cells, fibroblasts, and epithelial cells [130, 150, 151]. Thus, IL-33 is highly expressed in the lungs of both mice and humans [152]. Similar to IL-1α, IL-33 can be released upon the loss of cell integrity [153, 154]. Upon binding to its orphan receptor serum stimulation (ST)2 of the TLR/IL1R superfamily of receptors (also known as IL-1RL1), IL-33 initiates the potential signaling pathway via NF-κB and MyD88 [155, 156]. Interestingly, regulatory T cells that express the IL-33 receptor, ST2, upregulate amphiregulin after IL-33 stimulation, and this mechanism has been implicated recently in epithelial tissue repair after viral infection of the lung [157]. Again similar to IL-1α, IL-33 does not require processing to maintain its activity [158]. However, IL-33 can be cleaved to a mature and more proinflammatory form [159]. Interestingly, unlike other DAMPs, i.e., HMGB1 and IL-1α, nuclear IL-33 represses gene expression, and its nuclear localization alters the subcellular localization of IL-33, thereby regulating its extracellular functions and affecting immune homeostasis [160, 161]. On the other hand, comparable to other alarmins, posttranslational modification of IL-33 via its oxidation can regulate its activity, and thereby restrict its inflammatory function to the local environment [130, 162]. IL-33 plays an important role in inflammatory and autoimmune diseases such as asthma, inflammatory bowel disease, or autoimmune hepatitis by influencing neutrophilic infiltration into tissues, modulating the type 2 immune response, including Th2 cells, mast cells, eosinophils, basophils, and group 2 innate lymphoid cells [158, 163–166]. The relevance of IL-33 processing in a pathological environment is still not clear, but it is evident that an inflammatory microenvironment can exacerbate disease-associated functions of IL-33 via generating more active mature form [159].

Recently, Liu et al. [167] have shown that the sensitivity of IL-33-deficient mice to bleomycin-induced ALI and that this phenotype was reversible by complementation with rIL-33. The authors conclude that IL-33 is a crucial local factor necessary to control and limit the early inflammatory response after chemically induced ALI [167]. On the other hand, depending on the context of certain infections, IL-33 was either protective or even deleterious, because of its either beneficial role in the resolution of inflammatory processes, or detrimental function in aggravating inflammation [130, 154, 168]. Interestingly, IL-33 reduced mortality in septic mice due to increased neutrophil influx into the peritoneal cavity and thereby enhanced bacterial clearance as compared to controls [169]. The data are significant, since they show that IL-33 reduced systemic but not local proinflammatory responses, and thereby limited the sepsis-induced systemic inflammation [169]. Under certain conditions of chronic inflammation, IL-33 was deleterious during the development of, e.g., asthma, or in the context of chronic obstructive pulmonary disease (COPD) and arthritic joint inflammation [170–172]. The role of IL-33 in traumatic setting is completely elusive. However, there are some studies dealing with IL-33 during injury to the central nervous system (CNS) or spinal cord. It has been shown that IL-33 was highly expressed in white matter in the CNS, where it colocalized with oligodendrocyte markers as well as with astrocyte markers in gray matter [173]. Immediately after CNS injury IL-33 was released from damaged oligodendrocytes, acting on local astrocytes and microglia to induce chemokines critical for monocyte recruitment [173]. Mice lacking IL-33 had impaired recovery after CNS injury, which is associated with reduced myeloid cell infiltrates and decreased induction of M2 genes at the injury site [173]. Wicher et al. (2017) used samples from human TBI microdialysate, tissue sections from human TBI, and mouse models of central nervous system injury and found that expression of IL-33 in the brain was elevated from nondetectable levels, reaching a maximum after 72 h in both human samples and mouse models [174]. The authors have shown that astrocytes and oligodendrocytes were the main producers of IL-33 [174]. Post-TBI brains of mice deficient in the IL-33 receptor, ST2, contained fewer microglia/macrophages in the injured region as compared to the wild-type controls and had an altered cytokine/chemokine profile in response to injury [174]. Taken together, these observations indicate that IL-33 plays a role in neuroinflammation with microglia/macrophages being cellular targets for this interleukin post-TBI. Other in vivo experiments have shown before that after traumatic spinal cord injury (SCI) administration of recombinant IL-33 turned beneficial by significantly decreasing tissue loss, demyelination and astrogliosis in the contused mouse spinal cord, finally resulting in dramatically improved functional recovery [175]. Taken together, the recent findings indicate that IL-33 plays a role in response to TBI and drives chemokines that recruit monocytes and polarize macrophages toward an M2 phenotype, thereby potentially protecting neurons from further damage and promoting recovery after CNS injury [176]. Maybe a role of IL-33 as a potential SAMP should be carefully considered.

### S100A proteins

S100 proteins or calgranulins are a family of low molecular weight calcium-binding homodimeric proteins, consisting of at least 25 distinct members with a large variety of intracellular and/or extracellular functions [130]. They are localized intracellularly in the cytoplasm, and here, they interact with various effector molecules to regulate cell proliferation, differentiation, migration, energy metabolism, scavenging of reactive oxygen species (ROS) and nitric oxide, calcium homeostasis, inflammation, apoptosis, transcriptional regulation as well as DNA repair and others [16, 130]. S100 proteins are mainly expressed in cells of myeloid origin, predominantly in neutrophils and are induced in several cell types which mediate inflammatory responses and recruit inflammatory cells to sites of tissue damage [177]. These small proteins are known to be either passively released from damaged cells or actively secreted from activated cells, and they have been detected in various body fluids, such as serum, urine, sputum, cerebrospinal fluid, and feces of patients with cancer, inflammatory and autoimmune disease, or cardiovascular complications, atherosclerosis or stroke [130, 178–183]. Upon their release, they act as alarmins via their interaction with different receptors to orchestrate innate and adaptive immune responses [130, 184]. As example, S100A8, S100A9, and S100A12 family have been found extracellularly at high concentrations in inflamed tissue, where they exert their proinflammatory effects via binding to RAGE, TLR4 or upon interaction with other receptors [177, 185]. The signaling pathways trigger the activation of kinases including p38 MAPK, ERK1/2, and again transcription factor NF-κB [186–188]. S100A1 released from damaged cardiomyocytes during myocardial infarction triggers TLR4-dependent proinflammatory responses, leading to induction of myocardial damage [189]. Several studies have reported an increase in S100A8/A9 levels in sepsis [190–192]. However, certain anti-inflammatory properties in case of, e.g., S100A8 and S100A9 have been reported as well [193, 194].

With specific regard to traumatic injury, it has been demonstrated that S100B in CSF and plasma increases after injury and, even more, that the increasing levels were negatively correlated to outcome from human TBI [195–197]. It was shown that S100B can counteract and reduce some negative cellular consequences of injury [184, 196, 198]. However, more recent studies imply the negative role of S100B in TBI and represent that S100B in CSF, as an astrocytic protein specific to the central nervous system and a useful marker in outcome prediction for TBI [199, 200]. Thus, S100 s protein have been established as useful marker for inflammatory diseases and blood brain barrier damage, which plays an important role in the development and recovery of normal CNS after injury [201–203]. Recently, results of a meta-analysis showed a significant difference in S100B levels between survived and died subjects with head injuries on overall follow-up timeline, during treatment, or 6 months with an average threshold value that varies according to the study method used [203]. In terms of other S100 proteins and nervous injury, following peripheral nerve injury an immediate acute immune response distally and proximally to the lesion site with rapid transcriptional activation of S100a8 and S100a9 genes resulting in S100A8/A9 hetero- and homodimers has been observed [204]. The subsequently provoked release of chemokines and cytokines by activated Schwann cells induced an initial chemotactic gradient, which was responsible for the transmigration of hematogenous immune cells toward the traumatized site of the injured nerve [204]. Also after severe burn injury, S100A8 and S100A9 levels in bloodstream were correlated with poor outcome [205]. These proteins were found to be increased in a biphasic manner with the early peak at initial presentation representing the innate response to injury [205]. Similar to HMGB1, S100A8 and S100A9 levels in the bloodstream increase early in the acute phase of trauma or brain injury. Thus, although large number of studies deals with neurological trauma, there is some evidence that other S100 protein, i.e., S100B is also elevated in patients with major extracranial trauma [203, 206–208]. S100B concentrations upon admission correlated positively with injury severity and decreased survival in major trauma patients, observations which were independent of head injury [206, 208]. In general, circulating levels of S100B in severely injured trauma patients were increased as compared to healthy volunteers [206]. Even more, S100B levels correlated with systemic levels of sE-selectin and von willebrand factor, which are indicating endothelial cell injury [206, 209]. Further mechanistical analyses of human endothelial cells transfected with S100B uncovered an increase in apoptosis and levels of proinflammatory cytokines IL-6 and IL-8 [206]. Thus, S100B apparently correlates with endothelial damage indicating important pathomechanistical influence of S100B in the recovery from trauma. However, it cannot be ruled out that increased S100B after soft tissue trauma might occur due to peripheral nerve injury. On the other hand, currently a protective role of certain members of the S100 family as briefly addressed above cannot be ruled out. Notably in a study on human trauma victims, human blunt trauma survivors were seen to produce higher levels of S100A8 and S100A9 than non-survivors, and a preliminary screen suggested that interferon-γ inducible protein 10 (CXCL10) was significantly increased in monocytes/macrophages stimulated with S100A8/S100A9 [210]. Thus, heat shock protein (HSP)70, S100A8/S100A9 DAMPs appear to exert a protective role after trauma. This remains to be further elucidated in future.

### Histones

Other critical members of DAMPs are the circulating extracellular histones which act as clinically relevant mediators of multiple organ injuries [211, 212]. These nucleoproteins enable DNA compaction into nucleosomes, thereby contributing to the structural organization and stability of chromatin. As they localize in the nucleus, their release into the extracellular space, as free histones, DNA-bound histones (nucleosomes), or part of NETs, has been recently recognized as candidate of the DAMP family, and as such in all three forms, they can be detected in serum after significant cellular death which is occurring in infective or sterile SIRS [213, 214]. Their extracellular activity has a long history, while more than 50 years ago the first observations uncovered the antibacterial potential of these molecules [215, 216]. Since Xu et al. [217] have demonstrated that extracellular histones released in response to inflammatory challenge contributed to endothelial dysfunction, organ failure and death during sepsis, the mechanisms of histone-mediated injury in certain organs have been extensively studied. Clinically, elevated levels of circulating histones and histone–DNA complexes were associated with the incidence of MOF, disseminated intravascular coagulation, cardiac injury, arrhythmia, and ventricular dysfunction in patients with sepsis [218, 219]. Therefore, the extracellular histone also acts as an immunothrombotic agent. Furthermore, pharmacological targeting by antibodies to histones or by activated protein C (APC) reduced the mortality of septic mice [217]. Thus, the authors proposed extracellular histones as potential molecular targets for therapeutical treatment of sepsis and other inflammatory diseases.

Pathomechanistically, histone administration resulted in neutrophil margination, vacuolated endothelium, intra-alveolar hemorrhage and macro- and microvascular thrombosis in vivo [217]. Histones mainly bind and activate TLR, e.g., TLR2, TLR4 or TLR9 on various cells, and similar to other DAMP, they subsequently trigger the inflammatory response [213, 220, 221]. This has been confirmed in vivo, by showing that histone administration led to death after a dose-dependent aggravation of multiple organ injury in mice [211]. The causative effects for organ injuries were histone-driven endothelial damage, and the associated release of HMGB1 [211]. The authors concluded that extracellular histones induce multiple organ injury in two progressive stages via direct endothelia disruption, and the subsequent release of other DAMPs [211]. Circulating levels of histones were significantly increased after severe non-thoracic blunt trauma in patients [222]. Furthermore, enhanced histone levels positively correlated with severe complications such as the incidence of ALI, and dismal prognosis, as well as with markers of endothelial damage and coagulation activation [222]. The harmful impact of histones on endothelia has been confirmed in vitro and in vivo demonstrating that histones directly damaged endothelial cells, stimulated cytokine release (e.g., TNFα, IL-6, and IL-10), induced NET formation and MPO release [222]. An anti-histone antibody reduced the harmful changes and protected mice from histone-induced lethality [222]. Also in a large in vivo model of polytrauma, increased extracellular histone levels could have been linked to cardiac dysfunction after porcine multiple trauma [223]. Thus, histone release plays an important pathological role in trauma-associated injuries [222, 223].

### Heat shock proteins

So far, only a few studies have investigated the relation of HSP to trauma-induced tissue damage [224]. Heat shock or stress response is a highly preserved cell response to injury which is actually a cell defense mechanism characterized by an increased expression of heat shock or stress proteins. HSP constitutes a group of proteins that primarily act as molecular chaperones in the cytosol which are constitutively expressed by all cells. HSPs are essential for significant cell processes such as protein folding, protein protection from denaturation or aggregation, and facilitation of protein transport through membrane channels [225, 226]. Thus, their specific elevated expression during the heat shock response stands for their essential role in protecting cells from stress, and preparing them to survive under environmental challenges [227]. HSPs are induced by a variety of cellular stress factors, including hypo- and hyperthermia, UV radiation, pathogens, and other forms of stress [16, 227, 228]. HSP family members are named according to their molecular mass [229]. Although HSPs were considered for some time as intracellular molecules that could only be released from necrotic cells by a passive mechanism [230], meanwhile it is known that they may be released by non-necrotic cells via an active mechanism including the non-classic protein release pathway, through which HSPs are released both as free proteins and within highly immunogenic exosomes [231]. It has been under discussion, if HSP in general constitute DAMPs, or if the most widely studied HSP70 is rather an exception [224]. This is not easy to evaluate since very often contamination with immunogenic molecules, i.e., LPS or DNA, can generate false-positive responses and results.

Upon release, HSP70 can stimulate monocytes/macrophages, microglia, and dendritic cells via the TLR2 and 4 and cluster of differentiation (CD)14 pathways, subsequently leading to the activation of intracellular signaling pathways [229]. Next to TLR, HSP70 binds to CD36, CD40, CD91, siglec-5, and siglec-14, lectin-like oxidized low-density lipoprotein receptor 1, and scavenger receptor class A to induce pro- or anti-inflammatory responses on a range of cells, mainly those of the innate immune system with exception of T lymphocytes [232–234]. Notably in studies of cerebral ischemia, neurodegenerative diseases, and epilepsy, HSP70 reduces protein aggregates, intracellular inclusions, and apoptosis improving neurological outcomes [229, 235–237]. However, conflictive results are found regarding HSP functions. On the one hand increased levels of HSP27, HSP60, HSP70, and HSP90 in patients with infectious septic complications [238–240], and significant improvement in mortality, lung function, local and systemic inflammation in a mouse model of severe sepsis-induced ALI after therapeutic HSP90 inhibition have been observed [241]. On the other hand, in a CLP-induced sepsis model in HSP70.1/3 knockout mice, NF-κB binding/activation, TNFα and IL-6 in lungs and mortality were increased [242]. Thus, HSP70 may confer protection from ARDS via acting at least partly through the NF-κB pathway and thereby reducing the proinflammatory cytokine response [242]. In line with these observations, HSP70 played a protective role in an age-dependent response to sepsis by preventing excessive gut apoptosis and both pulmonary and systemic inflammation [243]. Thus, multiple data are available suggesting that HSP are potent regulators of inflammatory events.

Severe trauma causes enhanced expression of HSP in polymorphonuclear leukocytes (PMNL) during the acute post-injury phase [244]. In comparison with healthy volunteers increased expressions of HSP27, HSP60, HSP70, and HSP90 in PMNL from trauma patients or patients with burn trauma were found, indicating that this enhanced expression of HSP may regulate PMNL functions [244, 245]. Concomitant with these changes was an increase in oxidative activity in PMNL, and markedly inhibited cell apoptosis after thermal injury [245]. Extracellular HSP60 release was observed within 30 min after trauma and correlated with the development of ALI [246]. Extracellular HSP have powerful immune properties by activating the classical complement pathway, participating in exogenous antigen processing and presentation, and showing immune reactivity to endogenous HSP [247]. Thus, HSP can also have an immunostimulatory effect and activate the host inflammatory response [224]. In mechanistical in vitro studies, it was shown that HSP60 caused the release of nitric oxide by macrophages [246]. Taken together with previously described in vivo observations, the authors suggest that the extracellular release of the immature HSP60 may be associated with traumatic cell necrosis, and could be involved in the release of NO by immune competent cells, subsequently inducing an activation of the local inflammatory response [246]. Similarly, systemic HSP72 levels were markedly elevated immediately after admission of severely traumatized patients to the emergency department as compared to healthy volunteers [248]. Interestingly, elevated initial HSP72 levels were associated with improved survival in severely traumatized patients, without showing any association to the overall injury severity [248]. Notably concerning HSP72 conflictive reposts are shown. Pittet et al. [248] reported that HSP72 was neither related to the incidence or severity of the inflammatory response nor to post-injury organ dysfunction.

HSP70 serum concentrations have been up to ten times higher immediately after injury in polytraumatized patients versus control subjects, levels that remained elevated until 48 h after the accident following a time kinetics concordant with that previously described [75, 248]. The magnitude of this increase was related to injury severity and prediction of secondary infection [249]. If HSP70 levels decreased in the period from 60 to 90 h after trauma, the patient had a better outcome as compared to those patients without a decrease in HSP70 levels [249]. The high extracellular concentration of HSP70 was even greater in patients with MODS [224]. Although the authors did not include follow-up of survival after hospital discharge and all patients except one survived during admission, the greater elevation of extracellular HSP70 in patients with MODS corroborates the proinflammatory character of this protein in severe trauma as reported by others as well, who also correlated HSP70 with survival and morbidity [250, 251]. In summary, these data support the hypothesis that HSP70 is produced as a danger signal to stimulate the immune systems of trauma patients [224, 252].

### Nucleic acids

All human cells contain nucleic acids as deoxynucleic acid (DNA), messenger ribonucleic acid (RNA), or mtDNA while mature erythrocytes may also retain some residual non-functional mtDNA [253, 254]. The endosymbiotic theory suggesting that mitochondria originate from bacteria, provide the cellular content of mtDNA. Usually, nucleic acids are released into the circulation after cell necrosis and nuclear destruction, but their active release has been reported as well [16, 255, 256]. This so-called extracellular cfDNA can be built up by either DNA or different species of RNA, and based on its origin, whether it is host or pathogen derived, it accounts for a DAMP or a PAMP, respectively [255]. TLRs are evolutionary conserved pattern recognition receptors (PRR) for detection of multiple microbial products including bacterial DNA and RNA, sense invading microbes and initiate a rapid immune response [257, 258]. However, adjacent to membrane-bound TLR3, TLR7, TLR8, TLR9, and RAGE, soluble mainly cytosolic receptors retinoic acid-inducible gene I, melanoma differentiation-associated protein 5, and cyclic guanosine monophosphate-adenosine monophosphate can recognize the cfDNA signals [255, 259, 260]. Intracellular mechanisms of mtDNA inflammation include inflammasome activation and stimulator of interferon gene pathway activation [261]. However, although these mechanisms have not been confirmed directly in the trauma setting, based on available scientific research plausible mechanisms exist [262]. The cfDNA released from mammalian cells and bacteria is a potent stimulator of the innate immunity. The ability of the cell to distinguish dangerous nucleic acids from safe counterparts may be determined by the unique cellular location of nucleic acid-sensing PRR [263–265]. In addition to proinflammatory activity, elevated plasma DNA and mtDNA as well as nDNA in patients with pulmonary embolism as compared to those patients with submassive pulmonary embolism or other diagnoses (pneumonia, myocardial infarction, thrombophlebitis, or normal lung scans) suggest that cfDNA may induce potent prothrombotic effects as well [266, 267]. NETs, which constitute a meshwork of DNA fibers comprising histones and antimicrobial proteins, stimulated thrombus formation both in vitro and in vivo, while the treatment with deoxyribonuclease (DNase) or anticoagulant heparin prevented NET-mediated thrombus formation [268]. Thus, cfDNA acts as potent immunothrombotic agents [265]. Increased levels of cfDNA in the form of nuclear DNA (nDNA), mtDNA, or NETs were found and correlated with detrimental outcomes in patients with sepsis [269, 270], cancer [271], autoimmune disease [272], cardiopulmonary bypass surgery [273], and solid organ transplantation [274].

As recently reviewed by Thurairajah et al. [262], clinicians have regarded DNA and mitochondria as intracellular structures, unrelated to the pathophysiology of trauma. Meanwhile, we are beginning to understand the large impact of cfDNA on posttraumatic inflammation. Release of cfDNA plays an important role in trauma [84, 256]. The group around Hauser [256] has shown that traumatic injury causes a release of mtDAMPs into the circulation, which have functionally important immune consequences. These DAMPs include formyl peptides and mtDNA as well, which can activate human polymorphonuclear neutrophils via TLR [256]. Briefly, tissue injury caused by trauma results in uncontrolled rupture of cell membranes and cell contents are spilled into the extracellular space [275, 276]. Adjacent to the release of mtDNA, numerous other DAMPs and ROS from damaged neighboring cells promote further necrosis and mtDNA release [50, 277]. As observed in traumatized patients, cf-mtDNA concentration positively correlated with injury severity, incidence of SIRS, and mortality [278–281]. Plasma levels of DNA were increased concomitant with the injury severity caused by trauma, and the concentration of mtDNA correlated not only with SIRS but also with the occurrence of ALI and MODS [256, 278, 280–282]. In vivo studies confirmed that hemorrhagic shock and trauma raised plasma mtDNA concentration [283]. The raised mtDNA concentration persisted throughout the 7 days of sampling. Not only traumatic insults but also surgical interventions result in tissue injury and can also increase cf-mtDNA release [284]. Also upon femoral reaming or cardiopulmonary bypass surgery, high quantities of mtDAMPs were identified [285]. Even more, the higher concentrations of mtDNA correlated with postoperative complications [273]. Interestingly, endotracheal tube placement in surgery has been associated with higher concentrations of mtDNA in throat lavage fluid, and a correlation between non-infected sore throat post-intubation and elevated throat lavage mtDNA concentration was found [286]. Although it appears that the inflammatory effects of mtDNA can be beneficial and harmful, in the trauma setting, the increase in cf-mtDNA associated with MOF proposes rather a harmful scenario of mtDNA-induced inflammation [80, 278, 287]. Interestingly, Prikhodko et al. [288] have demonstrated that the levels of circulating mtDNA significantly increased in trauma patients compared to those in healthy volunteers, but that purified mtDNA could not stimulate innate immune cells. These data suggest that the level of extracellular DNA in the blood may be a suboptimal marker for human disease. Development of new approaches to detect circulating extracellular DNA that actually activates innate immune cells would be beneficial. Although enormous research and efforts to develop therapeutic agents that neutralize cf-mtDNA are ongoing, there is still insufficient knowledge to including this into clinical management. Current goals are limited to the validation of cf-mtDNA as a biomarker of injury severity, and/or predictor of SIRS/MOF to improve the clinical management of patients.

### Adenosine triphosphate

Adenosine 5′ triphosphate (ATP), another DAMP originating from mitochondria, can be released passively due to tissue damage or actively via channels or vesicular exocytosis, contributing to the induction of inflammation by activation and recruitment of various inflammatory cells, e.g., macrophages, neutrophils, and dendritic cells [46, 50, 289–293]. Stimulation of virtually any mammalian cell type leads to the release of cellular ATP and autocrine feedback through a diverse set of different purinergic receptors [294]. Extracellular ATP signaling is transduced, e.g., via the purinergic receptor P2X7 with subsequent efflux of potassium ions, with following aggregation and activation of inflammasomes [290, 291, 294]. Thus, ATP gating of P2X7R with the subsequent assembly of the NLRP3 inflammasome/caspase-1 complex is critical for IL-1β maturation as well as its release [295]. Human neutrophils, which play an important role in tissue damage and repair, release ATP from the leading edge of the cell surface to amplify chemotactic signals and direct cell orientation and migration by feedback through P2Y2 nucleotide receptors [293, 296]. Thus, ATP release and autocrine feedback through P2Y2 and A3 receptors provide signal amplification, controlling gradient sensing and migration of neutrophils [293]. It was shown that systemic ATP impairs polymorphonuclear neutrophil functions by disrupting the endogenous purinergic signaling mechanisms that regulate cell activation and chemotaxis [297]. Based on their data, the authors suggest that removal of systemic ATP improves polymorphonuclear neutrophil function and host defenses, making this a promising new treatment strategy for sepsis [297].

In case of, e.g., overaggressive mechanical ventilation strategies, direct injury or cyclic deformation can release massive amounts of extracellular ATP from type I alveolar epithelial cells [298]. High levels of ATP may saturate ATP degradation resulting in increased alveolar ATP levels despite the ongoing enzymatic conversion to the immunosuppressive purine, adenosine [298]. The authors suggest that this sequence of events may be an important contributor to both pneumonia and clinical ALI.

### Complement factors

Exposure to traumatic or infectious insults results in an immediate activation of the complement cascade as major fluid defense system of innate immunity [48, 299]. As nicely described by Huber-Lang, the complement system acts as a master alarm system during the molecular danger response after trauma and significantly contributes to the clearance of DAMPs and PAMPs [48, 299]. Activation of complement in trauma patients has been verified by detection of elevated plasma C3a, C5a, and C5b-9 levels which correlate with disease severity [73, 300–305]. In septic patients, there is abundant evidence for complement activation and production of the anaphylatoxin C5a. In an experimental model of CLP-induced sepsis, interception of C5a or its receptors was shown to greatly improve survival in rodents, an effect that was associated with mitigation of the consumptive septic coagulopathy as well as reduction in the hyperinflammatory response, thereby reducing intensity of MOF, and septic shock [306, 307]. However, depending on the origin or extent of the damaged macro- and micromilieu, not only activation but also inhibition of the complement system leading to a maladaptive immune response and subsequent cellular and organ dysfunctions was observed [48, 299].

### Extracellular vesicles

Extracellular vesicles (EVs) comprise various small, membranous vesicles that are released from activated or dying cells. Presently, there are two distinct populations of EVs based on their size: exosomes (30–100 nm) and microparticles (100–1000 nm). Different types of EVs are generated by different mechanisms of biogenesis [308]. Exosomes form by inward budding of multivesicular bodies membranes, while microparticles and apoptotic bodies are generated by outward budding of plasma membrane [308]. This process requires cytoskeletal reformations and sequential assembly of the endosomal sorting complex on the multivesicular bodies membranes [309–311].

Upon release, EVs transfer their cargo by multiple mechanisms, such as endocytosis, phagocytosis, micropinocytosis, and membrane fusion [312, 313]. EVs carry the molecular signature of their origination cells including proteins, mRNAs, miRNAs (miRs), and lipids which make EVs effective cell-to-cell communicators [314–319]. Therefore, specific EVs from activated or dying cells can be used as biomarkers. For example, circulating EVs with liver-specific proteome markers and miRs are released from lipotoxic hepatocytes in nonalcoholic fatty liver disease [320, 321]. Recently, it was shown that damaged hepatocytes from alcoholic liver disease released a key source of EVs containing a specific microRNA “barcode” [322]. This EVs specific, i.e., microRNA “barcode” is detectable in the blood [320, 322, 323]. Next to their biomarker character, EVs are potent immune modulators. Dendritic cell-derived exosomes express major histocompatibility complex (MHC) I, MHC II, and costimulatory molecules, and they can induce antigen-specific T cell responses [324–326]. In case of cancer, it has been shown that exosomes and microparticles released from cancer cells contributed to the suppression of host immune surveillance, cancer progression and metastasis, and angiogenesis [327–329]. In various other diseases, graft-versus-host disease, chronic kidney disease, and acute radiation injury EVs have been proposed as therapeutic to modulate the inflammatory responses and tissue regeneration [330–332]. However, this remains to be considered carefully, since EVs themselves can express various procoagulants promoting the vascular thrombosis [333, 334]. Exosomes isolated from septic patients induced vascular dysfunction by inducing reactive oxygen species generation and endothelial cell apoptosis [335]. Inhibition of the exosome release prior to endotoxin challenge or CLP in mice significantly reduced the levels of circulating exosomes and diminished sepsis-induced cardiac inflammation, myocardial dysfunction, and mortality [336, 337]. Interestingly, increasing evidence is demonstrating that the positive effects of cell-based therapies may be mediated by exosomes released from the administered cells and that the corresponding microRNA cargo in these exosomes is largely responsible for the therapeutic effects [338].

With regard to traumatic injury, recently Eguchi et al. [339] have demonstrated that liver injury in alcohol-intoxicated trauma patients with severe injury was reflected by increased systemic EV numbers, their specific miR barcode, and the correlated increase in systemic inflammatory markers IL-6 and IL-33, with IL-33 being a marker of alcoholic liver disease. Interestingly, severely injured trauma patients without liver injury were found to have a reduced number of liver-derived EVs, no observed inflammatory infiltration, and reduced specific miR “barcode” [339]. Trauma/hemorrhagic shock causes a release of proinflammatory mediators into the mesenteric lymph that may trigger a systemic inflammatory response and subsequent organ failure. Recently, it has been shown that exosomes in post-shock mesenteric lymph were biologically active mediators of this inflammation and that they carry a distinct, proinflammatory protein cargo [340, 341]. Interestingly, a stimulation of the vagus nerve prevented the T/HS-induced changes in mesenteric lymph exosome protein [341]. Subsequently, Williams et al. [341] propose a novel mechanism by which the neuroenteric axis may limit the systemic inflammatory response after injury protein. Neuroinflammation is a response against harmful effects of diverse stimuli and participates in the pathogenesis of brain and spinal cord injury. The innate immune response plays an important role in neuroinflammation following CNS injury via activation of inflammasomes [342, 343]. Interestingly, exosomes derived from neurons can deliver short-interfering RNA into the CNS to significantly decrease inflammasome activation after injury [343]. Thus, exosomes offer a new therapeutic approach to deliver RNA-based drugs to block inflammation after CNS injury. Several studies have characterized EVs in blood collected after traumatic injury, demonstrating their levels, cellular origins, adhesion molecule expression, and procoagulant activity [344–348]. Blood from patients who developed sepsis after trauma increased levels of platelet microparticles and platelet-leukocyte aggregates as compared to healthy controls were detected [344]. Kuravi et al. [349] have shown that a significant increase in plasma EVs after severe traumatic injury had procoagulant and proinflammatory effects that may influence outcomes. Thus, EVs as DAMPs may play dual roles in tissue repair and damage.

### Other DAMPs

Several other DAMPs playing important roles in the initiation and regulation of immune responses as well as in the post-injury regeneration have not been addressed here. Of those, notably other mitochondria-derived DAMPs including mitochondrial formyl peptides, heme, and cytochrome (Cyt)C have not been described [46].

Heme, a tetrapyrrole containing a central iron ion, is synthesized in the mitochondria and constitutes a versatile signaling molecule controlling the activities of diverse regulators ranging from transcription factors to MAP kinases [350]. Upon severe trauma, this DAMP can bind to TLR4 and trigger a number of downstream stress response pathways, and it was suggested to play a central role in the pathogenesis of severe sepsis [351].

CytC which is also released from dying cells during cellular trauma can act as a danger signal in the extracellular space. In experiments on rabbits, oxidative injury to mitochondria has been demonstrated to be associated with high mitochondrial expression of CytC [352]. In studies on a burn model in mice, circulating CytC levels were found to be elevated eightfold within 3 h post-injury and remained elevated at 24 h [353].

Upon destruction of the red cells in the blood vessels, a significant quantity of hemoglobin and other contents of these cells are released into the circulation [354]. In case that this cell-free hemoglobin is not neutralized to its inert, non-toxic form by its scavenger proteins, significant damage in the vascular, perivascular and endothelial spaces occurs [354, 355].

Extracellular cyclophilin A (CypA) is a DAMP that has been associated with rheumatoid arthritis, liver injury and severe sepsis as well [356–358]. It can act as a chemotactic agent for inflammatory cells via the CD147 receptor, and it can directly stimulate the proinflammatory response. However, in an in vitro shock tube model of blast TBI with human neuroblastoma cells, a potentially neuroprotective mechanism involving released CypA has been proposed, since the accumulation of CypA in the culture medium after repeated blast exposures supported the hypothesis that extracellular CypA-mediated neuroprotection [359]. Post-exposure treatment of the cells with purified recombinant CypA confirmed this protection against blast-induced neuronal injury [359].

Uric acid can be released from injured cells as other DAMPs as well [360]. Inside the cells, it is soluble, but it precipitates to monosodium urate microcrystals in its extracellular form, exerting inflammatory properties, as evident by its accumulation in tissues and gout [49, 361]. Uric acid crystals act via inflammasomes, resulting in the production of active proinflammatory cytokines IL-1β and IL-18 and neutrophilic influx [362]. Elevated levels of serum uric acid correlated with early acute kidney injury after severe burns [363]. The authors propose uric acid-related aberrant inflammation to be one of the pathogenic factors [363].

## Recognition, signaling and cellular response

The “danger model” was proposed as an alternative to the “self versus non-self recognition model” [364, 365]. Briefly, the immune system discriminates not only between self and non-self, but also between safe and dangerous as well, as clinically proved by Walter Land and presented by Polly Matzinger in the “Danger Model,” expanding the work of Janeway and others [70, 71, 364–367]. This complex response to stress employs numerous equivalent or comparable components of PAMPs, DAMPs, or newly introduced SAMPs, which can be found in most vertebrates, invertebrates, and even plants [23, 366–369]. These molecules are sensitized and recognized via PRR mediating very potent immune responses [370, 371]. Several classes of PRR have been identified so far, including the most prominent group of toll-like receptors (TLR), nucleotide oligomerization domain (NOD)-like receptors (NLR), members of the c-type lectin receptors (CLR) like mannose binding lectine (MBL) or receptor for advanced glycation end products (RAGE) among others [187, 370, 372, 373].

### TLRs

Among TLRs, membrane-bound TLRs recognize microbial components and environmental danger signals, such as lipopolysaccharide (LPS) of gram negative, lipoteichoic acid, or peptidoglycan of Gram-positive bacteria, or even liporabinomannan of mycoplasma, and, e.g., endogenous HMGB proteins or HEME [370, 374–378]. Intracellular TLRs recognize predominantly nucleic acids derived from bacteria and viruses, such as single- or double-stranded RNA from viruses, unmethylated CpG motifs, or purine analogs and other components of cellular stress [370]. PRR signaling is intracellularly transduced, e.g., via MAPK pathways to nuclei, with subsequent activation of diverse transcription factors, including NF-κB, and thereby induced a cellular responses [379, 380]. The cellular responses include, i.e., expression of cytokines or adhesion molecules to accelerate inflammation and diapedesis of the immune effector cells [380]. Interestingly, in a feedback loop, those inflammatory mediators themselves can induce, e.g., DAMPs to potentiate inflammation [381].

The cell surface receptor RAGE belongs to the immunoglobulin superfamily and was first described in 1992. Interestingly, RAGE is a multiligand receptor that can bind structurally diverse array of several molecules, including DAMPs like HMGB1, but also S100 family members and amyloid-β proteins [382–384]. RAGE is predominantly involved in the recognition of endogenous molecules released in the context of infection, chronic inflammation, or physiological stress, and its activation has been confirmed in various diseases, including sepsis and cardiovascular disease among others [187, 385–387]. Since RAGE shares numerous TLR ligands, its activation can induce numerous cellular signaling pathways, which among other transcription factors modulate NF-κB, activator protein-1, or signal transducers and activators of transcription-3 [187, 388–390]. In general, RAGE triggers a proinflammatory response, generation of nitric oxide, and several adhesion molecules but also RAGE itself causing and enhancing a continuous inflammatory response [389, 391–394].

### NLR

Adjacent to TLRs, NLR sensitizes signals of cellular stress, such as ATP-induced activation of P2X7 channels and the efflux of potassium ions, host cell-free nuclear DNA, ROS as well as bacterial peptidoglycans, crystalline material, peptide aggregates, bacterial toxins and numerous others [395–397]. As part of the multiprotein inflammasome complexes, they mediate the processing of biologically inactive precursors of, e.g., IL-1β or IL-18 into their respective bioactive forms, which furthermore promote pyroptosis, a specific form of cell death [396, 398–402]. A direct link for the NLR sensor molecule-mediated inflammasome activation via binding of either a PAMP or a DAMP is still under discussion [397, 402]. The proposed classical mechanism implies two signals, the first one mediated by a TLR [403, 404], and in parallel, the potential DAMP signal for inflammasome induction by, e.g., potassium influx or binding of ATP to P2X7 [126, 402–407]. Apart from activating certain cytokines, the inflammasome complex induces cell lysis in form of pyroptosis, which has been introduced in 2001 [408]. Pyroptosis describes the well-orchestrated lysis of the cell, which is initiated by an inflammasome activation and consecutive formation of caspase-1-dependent pores of 1–2 nm width [408]. As a consequence, cells are prone to swelling and lysis, but also to the release of intracellular molecules, which act as DAMPs, e.g., HMGB1 or ATP, which are described above. Briefly, pyroptosis is an important mode of cell death in DAMP-mediated enhancing and spreading of the inflammatory response, which mainly affects the cells of the myeloid lineage but also occurs in epithelial, endothelial cells and neurons.

### CLR

CLR has been mainly described in the context of binding to PAMPs [409]. There are two groups of transmembrane CLR, like dectin-1 or dectin-2 subgroups, and a group of soluble CLR including MBL, which comprise this large family of receptors that are expressed on most cell types including macrophages and dendritic cells [409, 410]. The signaling pathways are either mediated directly via, e.g., NF-κB, or modulated signaling by TLR, triggering cellular phagocytosis, maturation of dendritic cells, chemotaxis, respiratory burst, and cytokine production [410]. More than 10 years ago, Yamasaki et al. [411] found that macrophage-inducible c-type lectin (Mincle) senses nonhomeostatic necrotic dead of cells due to a component of small nuclear ribonucleoprotein, spliceosome-associated protein 130 (SAP130), and induces thereby the production of inflammatory cytokines and chemokines to potentiate the neutrophilic infiltration of damaged tissue. The endogenous Mincle ligand SAP130 was confirmed as a danger signal, which can be released by damaged cells, thereby activating inflammatory responses including inflammasome activation [412].

## Conclusions

This review discusses only a few of the currently discussed DAMPs that play important roles in the inflammatory or regenerative responses upon trauma. Although the provided list is certainly both incomplete and provides only a limited overview to the concept of trauma-induced DAMPs, it remains evident that due to the bivalent character and often pleotropic effects of a DAMP, its functions in the posttraumatic inflammation and regeneration still remain to be studied in detail. However, the new insights into the contribution of DAMPs to the sepsis-like inflammation and immune paralysis-like states that can be observed in severely injured patients will be helpful in further specifying the clinical picture presented by, e.g., polytrauma. Considering the “SIRS and CARS paradigm,” and the terms mixed antagonist response syndrome and persistent inflammation, immunosuppression and catabolism syndrome, the role of these injury-induced activating and suppressing molecules clearly marks their predominant role in posttraumatic pathologies. It is indisputable that both systemic as well local presence of DAMPs is obligatory for the immune response upon traumatic insult. Yet, adjacent to their ability to be used as biomarkers to indicate or monitor disease or injury severity, and for eventual improving the timing of secondary surgery, they remain either negative or positive contributing factors for the disease development. In polytrauma, there is tentative evidence of a model suggesting that, simultaneously or somewhat retardedly with uncontrolled overshooting emission of proinflammatory DAMPs in excess (resulting in hyperinflammatory SIRS and MOF), uncontrolled overshooting generation/emission of SAMPs in excess takes place. Those “suppressing molecules” may lead to an exaggerated and long-lasting CARS associated with a stage of immunosuppression—still aimed at maintaining tissue homeostasis. Considering this scenario, it certainly remains to be further discussed if the biologically natural attempt of the innate immune defense system to reach homeostasis via emission of SAMPs in case of severe injury-induced SIRS potentially and tragically ends up with the creation of an overshooting anti-inflammatory/proresolving and immunosuppressive milieu rendering the patient susceptible to secondary life-threatening, often lethal infections. Although the data from SAMPs involved in polytrauma are sparse and, though, the various suppressing molecules are not systemically investigated, there is preliminary evidence suggesting a role of this class of suppressing DAMPs in posttraumatic inflammation. Therefore, increased knowledge exploring the dual role of DAMPs and the interplay with SAMPs after trauma, and with their nuclear functions, transcriptional targets, release mechanisms, cellular sources, and other functions and interactions in traumatized patients is warranted. Adjacent to in vivo and in vitro studies, and furthermore, based on some contradictory findings, which often simply originate from differences between the immune system of animals and human, further clinical research is necessary to resolve this puzzle.

## Abbreviations

AM: Alveolar macrophage
APC: Activated protein C
ARDS: Acute respiratory distress syndrome
ATP: Adenosine triphosphate
BAL: Bronchoalveolar lavage fluid
CARS: Compensatory anti-inflammatory response syndrome
CD: Cluster of differentiation
cfDNA: Cell-free DNA
COPD: Chronic obstructive pulmonary disease
CLP: Cecal ligation and puncture
CLR: C-type lectin receptor
CNS: Central nervous system
CSF: Cerebrospinal fluid
CXCL: Chemokine (C-X-C motif) ligand
CypA: Cyclophilin A
CytC: Cytochome C
DAMP: Damage-associated molecular pattern
DIC: Disseminated intravascular coagulation
DNA: Deoxyribonucleic acid
EV: Extracellular vesicles
HMGB: High-mobility group box
HSP: Heat shock protein
ICU: Intensive care unit
IL: Interleukin
IL-1R: Interleukin-1 receptor
IS: Immunosuppression
LPS: Lipopolysaccharide
MAPK: Mitogen-activated protein kinase
MBL: Mannose binding lectine
MHC: Major histocompatibility complex
Mincle: Macrophage-inducible C-type lectin
MIP: Macrophage-inflammatory protein
miRNA: MicroRNA
MODS: Multiple organ dysfunction syndrome
MOF: Multiple organ failure
MPO: Myeloperoxidase
mRNA: Messenger RNA
mtDNA: Mitochondrial DNA
MyD88: Myeloid differentiation primary response 88
NET: Neutrophil extracellular traps
NF-κB: Nuclear factor “kappa-light-chain-enhancer” of activated B cells
NLR: NOD-like receptor
NLRP: Pyrin domain-containing nucleotide oligomerization receptor (NLR)
NOD: Nucleotide oligomerization domain
nRNA: Nuclear RNA
PAMP: Pathogen-associated molecular pattern
PMNL: Polymorphonuclear leukocytes
PRR: Pattern recognition receptor
RAGE: Receptor for advanced glycation end products of proteins
ROS: Reactive oxygen species
RNA: Ribonucleic acid
SAMP: Suppressing/inhibiting inducible DAMP
SAP130: Spliceosome-associated protein 130
SCI: Spinal cord injury
SIRS: Systematic inflammatory response syndrome
ST2: Orphan receptor serum stimulation 2
TBI: Traumatic brain injury
Th: T-helper cell
TLR: Toll-like receptor
TNF: Tumor necrosis factor
WT: Wild type
